# Outcomes in Cardiogenic Shock Patients with Extracorporeal Membrane Oxygenation Use: A Matched Cohort Study in Hospitals across the United States

**DOI:** 10.1155/2018/2428648

**Published:** 2018-01-22

**Authors:** Rayan El Sibai, Rana Bachir, Mazen El Sayed

**Affiliations:** ^1^Department of Emergency Medicine, American University of Beirut Medical Center, Beirut, Lebanon; ^2^Emergency Medical Services and Prehospital Care Program, American University of Beirut Medical Center, Beirut, Lebanon

## Abstract

**Background:**

ECMO is increasingly used for patients with critical illnesses. This study examines ECMO use in patients with cardiogenic shock in US hospitals and associated outcomes (mortality, hospital length of stay, and total hospital charges).

**Methods:**

A matched cohort retrospective study was conducted using the 2013 Nationwide Emergency Department Sample. Cardiogenic shock visits were matched (1 : 1) and compared based on ECMO use.

**Results:**

Patients with ECMO (*N* = 802) were compared to patients without ECMO (*N* = 805). Mortality was higher in the ECMO group (48.9% versus 4.0%, *p* < 0.001). Visits with ECMO use also had higher average hospital charges ($580,065.8 versus $156,436.5, *p* < 0.001) and average hospital LOS (21.3 versus 11.6 days, *p* < 0.001). After adjusting for confounders, mortality (OR = 8.52 (95% CI: 2.84–25.58)) and charges (OR = 1.03 (95% CI: 1.02–1.05)) remained higher in the ECMO group, while LOS was similar (OR = 1.01 (95% CI: 0.99–1.02)).

**Conclusions:**

Patients with cardiogenic shock who underwent ECMO had increased mortality and higher cost of care without significant increase in LOS when compared to patients with cardiogenic shock without ECMO use. Prospective evaluation of this observed association is needed to improve outcomes and resources' utilization further.

## 1. Introduction

Extracorporeal membrane oxygenation (ECMO) is a method of mechanical cardiorespiratory support used in critical cases, usually in intensive care units or emergency department (ED) settings [1]. Early evidence for ECMO efficacy was discouraging and the adoption of ECMO in the medical field was initially slow. A randomized controlled trial conducted in 1979 showed that ECMO did not increase long-term survival and resulted in a 90% mortality rate among adult patients with acute respiratory failure [2].

More recently and according to a recent study based on the Nationwide Inpatient Sample (2000–2011), ECMO use increased significantly (mainly after 2007) and was associated with an increase in healthcare associated costs including increased length of hospital stay (LOS) without improvement in survival [3]. The evidence for ECMO benefits was however becoming more evident especially after influenza H1N1 epidemic. Improved survival was documented with ECMO use among patients with acute respiratory distress syndrome (ARDS) [4–8]. This benefit extended to different clinical settings including the prehospital setting [9] and EDs [10] and to other clinical conditions such as near drowning [11], myocarditis [12], hypothermia [13], overdose [14], and pulmonary embolism [15]. Patients suffering from acute cardiac diseases, including cardiogenic shock, also had improved outcomes after ECMO use [16–20].

Cardiogenic shock is a critical condition characterized by low cardiac output and organ hypoperfusion with hypotension for 30 minutes and elevated left ventricular pressures [21]. Mortality rates for cardiogenic shock can reach up to 40% [22]. According to Extracorporeal Life Support Organization's data registry, cardiogenic shock was the most common cardiac indication for ECMO use in 2015 [23]. Indications for ECMO use usually include persons with severe, acute cardiac and/or respiratory failure who have failed to respond to conventional medical management [24]. A standard set of criteria for ECMO use for patients presenting with cardiogenic shock does not however exist. Cardiac indications for ECMO use typically include low cardiac output and hypotension despite adequate intervention (intravascular volume replacement, inotropic pharmacotherapy, and use of other forms of mechanical circulatory support) [25]. The guidelines for management of heart failure by The American College of Cardiology Foundation/American Heart Association also provide more detailed patient selection criteria for mechanical circulatory support such as ECMO. These criteria consist of “patients with LVEF < 25% and NYHA (New York Heart Association) class III-IV functional status despite guideline-directed medical therapy, including, when indicated, cardiac resynchronization therapy, with either high predicted 1- to 2-year mortality (e.g., as suggested by markedly reduced peak oxygen consumption and clinical prognostic scores) or dependence on continuous parenteral inotropic support” [26].

ECMO has also been shown to be an effective method for supporting hemodynamics in patients with cardiogenic shock due to myocarditis, myocardial infarction, and postcardiotomy [12, 27, 28]. The impact of ECMO use on mortality remains however controversial in this subpopulation: Diddle et al. reported a survival rate to hospital discharge of 61% for patients with acute myocarditis with ECMO use [12]. Kim et al. described ECMO use for patients with cardiogenic shock postmyocardial infarction with reported survival rate to hospital discharge of 59.3% [27]. On the other hand, a high hospital mortality of 67% was reported for patients with refractory cardiogenic shock postcardiotomy [28].

The increasing evidence for ECMO use in cardiogenic shock is showing promise; however its impact is not clear, especially with its associated increase in cost of care and resource utilization. This study examined ECMO use and outcomes of patients with cardiogenic shock (mortality, hospital length of stay, and total hospital charges) in US hospitals.

## 2. Methods

### 2.1. Study Design and Setting

This matched retrospective cohort study used discharge data from the Nationwide Emergency Department Sample (NEDS) database. NEDS represents the largest all-payer ED database in the United States and is a Healthcare Cost and Utilization Project (HCUP) database that is sponsored by the Agency for Healthcare Research and Quality (AHRQ) [29].

NEDS combines both clinical and nonclinical variables from both national and state sources, specifically 947 hospitals that represent a 20% stratified sample of hospital-based EDs across 30 participating states in the US. HCUP recommendations and instructions were followed for data weighting using the following stratification variables: US Census region, urban-rural location, ownership, and teaching status of the hospital and trauma center designation [30].

An institutional review board exemption from the American University of Beirut was obtained for the use of this deidentified database. Additionally, data on any variable with size less than or equal to 10 were excluded in order to safeguard patients' privacy and as per HCUP requirements.

### 2.2. Available Data

NEDS provides data for the following variables: diagnoses and procedural information; demographic patient information; mechanism of injury, intentional harm, and severity of injury; admission and discharge status; payment source; healthcare expenses; and general hospital characteristics. Diagnoses are available as the International Classification of Diseases, Ninth Revision, Clinical Modification (ICD-9-CM) codes as well as an equivalent and more manageable number of clinically meaningful Clinical Classifications Software (CCS) codes [31].

**Figure 1:**
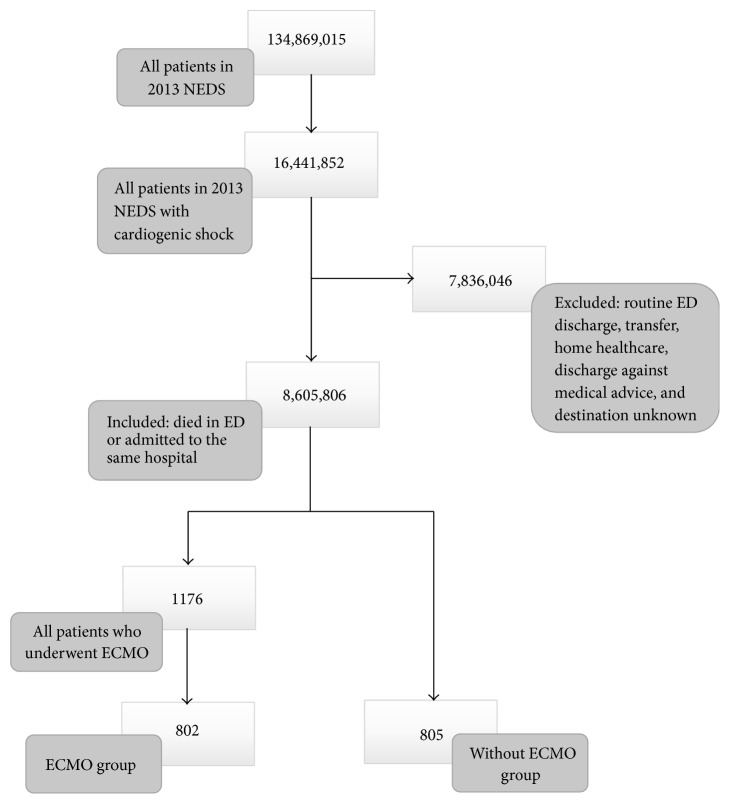
Flow chart of patients who met inclusion/exclusion criteria for the study population.

The following CCS codes were adopted from Maxwell et al. to select those presenting with cardiogenic shock: CCS 97, CCS 100, CCS 101, CCS 103, CCS 106, CCS 107, and CCS 108 [32] (a list of equivalent ICD-9-CM codes and variable classification is included as “Supplementary Material (available here)”). ECMO use was selected using the ICD-9-CM 3965 procedure code. Patients who were routinely discharged, transferred, discharged to home healthcare, or discharged against medical advice or whose destination was unknown were excluded from the study.  Figure 1 shows a flow chart of patients who met inclusion/exclusion criteria for the study population.

A group of patients with cardiogenic shock and reported ECMO use were randomly matched (1 : 1) with another group with cardiogenic shock without ECMO use. The following variables were used for matching: age (match tolerance = 2), sex, season of admission, whether admission day is a weekday or a weekend, presence of chronic conditions, Injury Severity Score (match tolerance = 1), primary expected payer, median household income, hospital urban/rural designation, and the four categories of procedure class (minor diagnostic, minor therapeutic, major diagnostic, and major therapeutic). A procedure is minor or major in terms of invasiveness and/or resource use based on ICD-9-CM procedure codes.

### 2.3. Statistical Analysis

Descriptive analysis of the study population was done using IBM-SPSS 24. Mean and associated 95% confidence interval (CI) were reported for continuous variables, and frequencies, percentages, and 95% CI were reported for categorical variables. A *p* value of <0.05 was used to denote statistical significance. HCUPnet, a free online query system based on data from HCUP, was also used to verify and confirm certain analyses.

The Rao-Scott chi-square test, a modified version of Pearson's chi-square test, was used to compare all variables between the two groups at the bivariate level. A logistic regression analysis was used for mortality, while multivariate linear regression was used for LOS and total hospital charge to examine their association with ECMO procedure (yes/no) in the matched data set, adjusting for significant variables. These variables included chronic conditions (infectious and parasitic disease; diseases of blood and blood-forming organs; mental disorders; diseases of the respiratory system; diseases of the genitourinary system; diseases of the skin and subcutaneous tissue; diseases of the musculoskeletal system; symptoms, signs, and ill-defined conditions; injury and poisoning; factors influencing health status; and contact with health services) and selected procedures (temporary and permanent tracheostomy; diagnostic bronchoscopy and biopsy of bronchus; incision of pleura; thoracentesis; chest drainage; other operating room Rx procedures on respiratory system and mediastinum; coronary artery bypass graft; percutaneous transluminal coronary angioplasty; other Operating Room heart procedures; other vascular catheterization, not heart; other non-Operating Room therapeutic cardiovascular procedures; respiratory intubation and mechanical ventilation; blood transfusion; conversion cardiac rhythm). Crude and adjusted odds ratios along with their corresponding 95% CI were calculated. To adjust for the NEDS survey design in developing estimates, the CSDESCRIPTIVES, CSTABULATE, and CSLOGISTIC procedures were used.

## 3. Results

A total of 134,869,015 weighted ED visits were available in 2013 in the NEDS database. Of those, 16,441,852 were visits that included cardiogenic shock as a diagnostic code. Only 1,176 weighted visits had ECMO procedure documented. After matching for the above-described variables, 802 visits with ECMO were successfully matched with 805 visits without ECMO.  Table 1 shows a list of matched characteristics and demographic variables. Patients who underwent ECMO had an average age of 49.9 years (95% CI: 47.1–52.5). They were mostly males (68.4%, 95% CI: 61.6–74.4). A higher frequency of visits (36.3%, 95% CI: 30.1–43.1) was noted to have a corresponding high household median income ($64,000 or more). More admissions occurred during weekdays (73.7%, 95% CI: 67.1–79.3). Patients underwent mainly major therapeutic procedures (ECMO included).

Table 2 shows characteristics and demographics that are significantly different between the two groups. While both groups were matched for presence of a chronic condition, the prevalence of subtypes of chronic conditions was different. Diseases of the cardiovascular system were most frequent in both ECMO and non-ECMO groups (100% and 96.8%).

The ED and inpatient procedures recorded for visits in both groups were also different. Only procedures that had significantly different frequencies between the two groups were included in Table 2 (total of 14 procedures). “Respiratory intubation and mechanical ventilation” was the second most frequently performed procedure for patients who underwent ECMO (56.9%, 95% CI: 49.9–63.6). In contrast, only 12.5% of patients in the group without ECMO underwent “respiratory intubation and mechanical ventilation.” The procedures used most often in this latter group were “other nonoperating room therapeutic cardiovascular procedures” (29.8%; 95% CI: 23.9–36.3). Two other procedures were more frequent in the group without ECMO use compared to the group with ECMO use; these were “percutaneous transluminal coronary angioplasty” and “amputation of the lower extremity.”

Table 3 shows main outcome differences between the two groups. Significantly higher mortality was noted in the group with ECMO use (48.9% versus 4%). Visits with ECMO use also had significantly higher average total charges incurred for both ED and inpatient services ($580,065.8 versus $156,436.50) as well as increased average LOS (21.3 versus 11.3 days). Lastly, Table 4 shows odds ratios (OR) of both primary and secondary outcomes before and after adjusting for all variables previously mentioned. Patients with cardiogenic shock and for whom ECMO was used had 8.52 higher likelihood of mortality when compared to those without ECMO use. ECMO use was also associated with higher charges (mean difference = $228,896.0 (standard error of the mean = 55403.8)) but no significant change in hospital LOS (mean difference = 0.8 (standard error of the mean = 4.9)).

## 4. Discussion

This study used a retrospective matched cohort design from large national US database of ED visits to examine clinical and financial impact of ECMO use in patients with cardiogenic shock. ECMO use was associated with significantly higher mortality and higher charges in this population.

The mortality rate in the group with ECMO use was 48.9%. This rate is lower than previously reported rates in studies for ECMO use in cardiogenic shock patients and in patients with other medical conditions (66.2% for years 2000–2007 and 63.7% for years 2007–2011) [32]. Survival related to ECMO use may be increasing over time due to improved equipment, more experience and larger caseloads, more specialized centers, more stringent patient selection, improved multidisciplinary team approach, or a combination of any or all the former factors.

The mortality rate in the cardiogenic shock group without ECMO on the other hand was strikingly low (4.0%). The study adopted CCS codes for cardiogenic shock that were previously used to define cardiogenic shock in studies originating from NEDS [32]. This low mortality rate may be related to the different proportions of cardiogenic shock etiologies. In fact, specific etiologies such as acute coronary syndrome are independent predictors of mortality, while other etiologies of cardiogenic shock are associated with lower mortality [22]. Etiologies other than acute coronary syndrome may be predominant in this study population. This variable was however missing in the dataset. Inclusion and exclusion criteria might also be different than other studies reporting higher mortality rates for patients with cardiogenic shock.

Patients who used ECMO in our study had a mean age of 49.9 years. A previous study by Maxwell et al. using NEDS (1998–2009) reported an overall mean age of 53.9 (±0.4) years and an average age of 48.9 (±0.8) for patients with cardiogenic shock with ECMO use [32]. The majority of people who underwent ECMO in our population were also males (68.4%). This is also comparable to findings by Maxwell et al. where 57.5% of patients in the cardiogenic shock group were males [32]. Other studies have also reported similar findings related to male gender and ECMO use in cardiogenic shock and this may be partly related to the higher prevalence of cardiac diseases among males [3].

Visits for cardiogenic shock with ECMO use had much higher charges than visits for cardiogenic shock alone. The average combined hospital and ED charge was $580,065.8 (95% CI: 482,527.3–677,604.2) per visit in the group with ECMO use compared to an average of $156,436.5 (95% CI: 123,792.1–189,080.9) per visit in the group without ECMO use. This average for charges per visit for cardiogenic shock with ECMO use is higher than what was previously reported in the literature during the years 1998–2009 ($344,009 (±$30,707)) [32]. ECMO associated costs are therefore on the rise. ECMO is a complicated procedure that is not easy to implement in a hospital and requires extensive resources and the formation of a comprehensive team of physicians, nurses, and staff [33]. The Extracorporeal Life Support Organization guidelines for ECMO implementation aim at maximizing efficiency and effectiveness of ECMO [33]. This initiative when coupled with advancements in ECMO machines and technology can reduce charges associated with ECMO use [34].

This study also examined the impact of ECMO use on hospital LOS. After adjusting for confounders, the increase in LOS was not significant. The average LOS of 21.3 days for visits with cardiogenic shock with ECMO is consistent finding from a previous study (19.9 days (2000–2007) and 22.6 days (2007–2011)) [3].

Our study has some limitations. One limitation stems from this study's inherent retrospective nature. Visits were identified using discharge diagnoses coding (CCS codes and ICD-9-CM codes) which is dependent on the quality of data, on the expertise and proficiency of the coder, and on the completeness of the patients' records. It is possible that some cases were not included because of coding deficiencies; however, NEDS has been shown to be one of the most robust and inclusive ED datasets available [35]. Additionally, both ICD-9-CM codes and CCS codes do not differentiate between venovenous and venoarterial ECMO. Venovenous ECMO provides respiratory support while venoarterial ECMO compensates for both the respiratory and the hemodynamic supports [25]. Cardiogenic shock is currently an indication only for venoarterial ECMO since it is characterized by cardiac insufficiency [25]. Future use of ICD-10-CM codes would mitigate this limitation.

Another limitation is related to missing important clinical variables from NEDS. Earlier cohort studies on venoarterial ECMO use among cardiogenic shock patients (ENCOURAGE and SAVE) have described significant clinical variables that impact outcomes [36, 37]. NEDS mainly collects administrative data and does not include specific clinical variables (e.g., SOFA score, pre-ECMO use hemodynamics and metabolic parameters, length of ECMO use, and indications for ECMO use) that reflect case mix or clinical severity. As a result, certain confounding variables such as clinical severity and critical nature of each visit were not controlled for. This study however used different methods (matching and logistic regression) to control for several variables considered to be proxies of clinical severity including specific major diagnostic and therapeutic procedures related to cardiac and pulmonary failure (e.g., temporary and permanent tracheostomy, incision of pleura, other Operating Room Rx procedures on respiratory system and mediastinum, coronary artery bypass graft, percutaneous transluminal coronary angioplasty, and other Operating Room heart procedures) as well as the presence of chronic medical conditions among different body systems.

Despite these limitations, this study examined the association between a resource intensive clinical intervention and clinical and financial outcomes using the largest ED database from the United States. Its findings are important for assessing expansion or reduction of clinical applications of ECMO and can be easily generalized to other similar acute care settings in the US.

## 5. Conclusion

Patients with cardiogenic shock who underwent ECMO were found to have increased mortality and higher cost of care without significant increase in length of stay when compared to patients with cardiogenic shock without ECMO use. Future research consisting of randomized clinical trials should prospectively evaluate this observed association between ECMO use in cardiogenic shock patients and outcomes in order to improve further patient care and resources utilization.

## Figures and Tables

**Table 1:** Matched variables for the study groups^a^.

	*Without ECMO*		*With ECMO*	
	Mean	(95% CI)	Mean	(95% CI)

*Age*	50.0	(47.7–52.3)	49.9	(47.1–52.5)

	*N*	% (95% CI)	*N*	% (95% CI)

*Injury Severity Score (0–15)* ^*a*^	800	99.4 (96.9–99.9)	796	99.3 (96.0–99.0)
*Sex*				
Male	551	68.5 (61.6–74.6)	548	68.4 (61.6–74.4)
Female	254	31.5 (25.4–38.4)	254	31.6 (25.6–38.4)
*Season of admission*				
Winter	199	24.7 (19.5–30.8)	204	25.5 (20.2–31.7)
Spring	195	24.3 (18.8–30.8)	185	23.0 (17.9–29.2)
Summer	203	25.3 (19.8–31.7)	202	25.2 (19.8–31.5)
Autumn	207	25.7 (20.1–32.3)	210	26.2 (20.9–32.3)
*Admission day*				
Monday–Friday	596	74.0 (67.6–79.6)	591	73.7 (67.1–79.3)
Saturday-Sunday	209	26.0 (20.4–32.4)	211	26.3 (20.7–32.9)
*Median household income* ^*b*^				
$1–$37,999	113	14.1 (10.0–19.6)	115	14.3 (10.2–19.7)
$38,000–$47,999	202	25.1 (19.7–31.4)	210	26.2 (20.6–32.7)
$48,000–$63,999	184	22.9 (17.6–29.2)	185	23.1 (17.8–29.4)
$64,000 or more	305	37.9 (31.6–44.7)	292	36.3 (30.1–43.1)
*Primary expected payer*				
Medicare & Medicaid	385	47.9 (41.0–54.9)	384	47.8 (41.0–54.7)
Private including HMO	384	47.8 (40.9–54.7)	383	47.8 (41.0–54.7)
*Chronic condition *	805	100	802	100
*Procedure class*				
Minor diagnostic	438	54.5 (47.9–60.8)	448	55.9 (49.1–62.4)
Minor therapeutic	725	90.1 (85.3–93.4)	722	90.1 (85.5–93.3)
Major therapeutic	800	99.4 (96.4–99.9)	796	99.3 (95.9–99.9)

^a^Per agreement with HCUP, certain categories *of primary expected payer*, *major diagnostic procedure class, and major trauma category Injury Severity Score* were omitted from the table due to a variable count less than 10. ^b^According to national quartile for patient ZIP Code derived from ZIP Code-demographic data obtained from Claritas.

**Table 2:** Unmatched variables between the two groups^a^.

	*Without ECMO*		*With ECMO*		*p value*
	*N*	% (95% CI)	*N*	% (95% CI)	
*Chronic condition body system indicator*					
Infectious and parasitic disease	207	25.7 (20.1–32.3)	315	39.3 (32.8–46.1)	0.004
Blood and blood-forming organs	257	31.9 (25.8–38.6)	519	64.8 (57.8–71.2)	<0.001
Mental disorders	264	32.8 (26.6–39.6)	128	16.0 (11.5–21.7)	<0.001
Circulatory system	779	96.8 (93.0–98.5)	802	100	0.014
Respiratory system	261	32.4 (26.2–39.3)	655	81.7 (76.0–86.4)	<0.001
Genitourinary system	343	42.6 (35.9–49.6)	580	72.3 (65.6–78.2)	<0.001
Skin and subcutaneous tissue	130	16.2 (11.7–21.9)	48	6.1 (3.5–10.3)	0.001
Musculoskeletal system	233	28.9 (23.1–35.5)	60	7.4 (4.7–11.7)	<0.001
Symptoms/signs/ill-defined conditions	307	38.1 (31.7–45.0)	604	75.3 (68.8–80.8)	<0.001
Injury and poisoning	285	35.5 (29.0–42.5)	478	59.6 (52.6–66.2)	<0.001
Health status/contact with health services factors	537	66.7 (59.9–72.9)	320	39.9 (33.6–46.5)	<0.001
*Procedures* ^*b*^					
Extracorporeal membrane oxygenation (ECMO)	0	0	802	100	<0.001
Tracheostomy: temporary and permanent	20	2.5 (1.0–5.9)	81	10.2 (6.6–15.4)	0.002
Diagnostic bronchoscopy and biopsy of bronchus	25	3.0 (1.4–6.5)	95	11.8 (8.2–16.8)	0.001
Incision of pleura; thoracentesis; chest drainage	27	3.3 (1.5–7.2)	82	10.3 (6.8–15.2)	0.008
Other Operating Room Rx procedures on respiratory system and mediastinum	16	2.0 (0.7–5.6)	154	19.2 (14.3–25.2)	<0.001
Coronary artery bypass graft (CABG)	29	3.7 (1.9–6.9)	118	14.8 (10.8–19.9)	<0.001
Percutaneous transluminal coronary angioplasty (PTCA)	185	23.0 (17.8–29.3)	109	13.6 (9.5–19.1)	0.015
Other Operating Room heart procedures	39	4.9 (2.8–8.4)	401	50.0 (43.0–57.0)	<0.001
Other vascular catheterization, not heart	189	23.5 (18.1–29.9)	288	35.9 (29.8–42.6)	0.006
Other non-Operating Room therapeutic cardiovascular procedures	239	29.8 (23.9–36.3)	110	13.7 (9.6–19.2)	<0.001
Amputation lower extremity	42	5.2 (2.8–9.6)	11	1.4 (0.4–4.6)	0.04
Respiratory intubation and mechanical ventilation	101	12.5 (8.7–17.8)	456	56.9 (49.9–63.6)	<0.001
Blood transfusion	118	14.7 (10.5–20.3)	195	24.3 (19.0–30.6)	0.014
Conversion cardiac rhythm	28	3.5 (1.7–7.1)	146	18.2 (13.6–24.0)	<0.001

^a^The following variables were not significantly different between cases and controls: chronic conditions of endocrine/nutritional/metabolic and immunity disorders, nervous system and sense organs, digestive system, and involving congenital anomalies; presence of injury diagnosis on record; or presence of unintentional injury. ^b^All procedures are based on Clinical Classification Software (CCS) procedures except ECMO which is based on the International Classification of Diseases, Ninth Revision, Clinical Modification (ICD-9-CM) procedure codes.

**Table 3:** Comparison of outcomes between the two groups.

	*Without ECMO*		*With ECMO*		*p value*
	*N*	% (95% CI)	*N*	% (95% CI)	

*Primary outcome*					
Survived	773	96.0 (92.5–98.0)	410	51.1 (44.4–57.8)	<0.001
Died in the ED/hospital	32	4.0 (2.0–7.5)	392	48.9 (42.2–55.6)	

	Mean	(95% CI)	Mean	(95% CI)	

*Secondary outcomes*					
Total charges^a^	$156,436.50	(123792.1–189080.9)	$580,065.80	(482527.3–677604.2)	<0.001
LOS^b^	11.6	(8.2–15.1)	21.3	(17.3–25.3)	<0.001

^a^Total charges in US dollars for combined ED and inpatient services. ^b^Length of hospital stay in days.

**Table 4:** Unadjusted and adjusted outcomes in the matched dataset.

	*Unadjusted*			*Adjusted* ^*a*^		
	Mean difference	Standard error	*p* value	Mean difference	Standard error	*p* value

Total charges^b^	$423,629.3	52,436.6	<0.001	$228,896.0	55,403.8	<0.001
LOS^c^	9.6	2.7	<0.001	0.8	4.9	0.870

	OR	95% CI	*p* value	OR	95% CI	*p* value

Died in the ED/hospital	23.2	11.1–48.4	<0.001	8.5	2.8–25.6	<0.001

^a^Above outcome adjusted for the following variables: significant chronic conditions (infectious and parasitic disease, diseases of blood and blood-forming organs, mental disorders, diseases of the respiratory system, diseases of the genitourinary system, diseases of the skin and subcutaneous tissue, diseases of the musculoskeletal system, symptoms, signs, and ill-defined conditions, injury and poisoning, factors influencing health status, and contact with health services), and selected procedures (temporary and permanent tracheostomy, diagnostic bronchoscopy and biopsy of bronchus; incision of pleura; thoracentesis; chest drainage, other Operating Room Rx procedures on respiratory system and mediastinum; coronary artery bypass graft, percutaneous transluminal coronary angioplasty; other Operating Room heart procedures; other vascular catheterization, not heart; other non-Operating Room therapeutic cardiovascular procedures; respiratory intubation and mechanical ventilation; blood transfusion; conversion cardiac rhythm). ^b^Total charges in US dollars for combined ED and inpatient services. ^c^Length of hospital stay in days.

## Abbreviations

ECMO: Extracorporeal membrane oxygenation
ED: Emergency department
ARDS: Acute respiratory distress syndrome
CS: Cardiogenic shock
NEDS: Nationwide Emergency Department Sample
HCUP: Healthcare Cost and Utilization Project
AHRQ: Agency for Healthcare Research and Quality
ICD-9-CM: International Classification of Diseases, Ninth Revision, Clinical Modification
CCS: Clinical Classifications Software
CI: Confidence intervals
LOS: Length of hospital stay.

## Supplementary Materials

Supplementary Materials A list of equivalent ICD-9-CM codes and variable classification.
