# Supplementary material for: Outcomes in Cardiogenic Shock Patients with Extracorporeal Membrane Oxygenation Use: A Matched Cohort Study in Hospitals across the United States
Source: Biomed Res Int. 2018 Jan 22;2018:2428648. doi: 10.1155/2018/2428648 (PMC5896328; doi:10.1155/2018/2428648)
Supplement: Supplementary Materials — A list of equivalent ICD-9-CM codes and variable classification. [file 2428648.f1.pdf]

<u>Supplementary Material.</u>

A list of Equivalent ICD-9 CM codes and variable classification

1. CCS category equivalent to ICD-9 CM:

- CCS: 97 equivalent ICD-9-CM codes: 03282, 03640, 03641, 03642, 03643, 07420, 07421, 07422, 07423, 11281, 11503, 11504, 11513, 11514, 11593, 11594, 1303, 3910, 3911, 3912, 3918, 3919, 3920, 393, 3980, 39890, 39899, 4200, 42090, 42091, 42099, 4210, 4211, 4219, 4220, 42290, 42291, 42292, 42293, 42299, 4230, 4231, 4232, 4233, 4238, 4239, 4250, 4251, 42511, 42518, 4252, 4253, 4254, 4257, 4258. 4259, 4290
- CCS: 100 equivalent ICD-9-CM codes: 4100, 41000, 41001, 41002, 4101, 41010, 41011, 41012, 4102, 41020, 41021, 41022, 4103, 41030, 41031, 41032, 4104, 41040, 41041, 41042, 4105, 41050, 41051, 41052, 4106, 41060, 41061, 41062, 4107, 41070, 41071, 41072, 4108, 41080, 41081, 41082, 4109, 41090, 41091, 41092
- CCS: 101 equivalent ICD-9-CM codes: 4110, 4111, 4118, 41181, 41189, 412, 4130, 4131, 4139, 4140, 41400, 41401, 41406, 4142, 4143, 4144, 4148, 4149, V4581, V4582
- CCS: 103 equivalent ICD-9-CM codes: 4150, 4151, 41512, 41513, 41519, 4160, 4161, 4162, 4168, 4169, 4170, 4171, 4178, 4179, V1255
- CCS: 106 equivalent ICD-9-CM codes: 4270, 4271, 4272, 42731, 42732, 42760, 42761, 42769, 42781, 42789, 4279, 7850, 7851
- CCS: 107 equivalent ICD-9-CM codes: 42741, 42742, 4275
- CCS: 108 equivalent ICD-9-CM codes: 39891, 4280, 4281, 42820, 42821, 42822, 42823, 42830, 42831, 42832, 42833, 42840, 42841, 42842, 42843, 4289

2. Variable Classification

Mortality, the main outcome of interest, was defined as dying in the ED or in the hospital setting.

Socio- Demographic variables:

- Age
- Sex
- Household median income quartiles for patient's ZIP Code was categorized into:
  - (1) $1 - $37,999; (2) $38,000 - $47,999; (3) $48,000 - $63,999; and (4) $64,000 or more
- Primary expected payer was categorized into:
  - (1) Medicare/Medicaid, (2) private including HMO, (3) self-pay, (4) no charge, and (5) other

Administrative characteristics

- Month of admission from (1) January to (12) December. To improve the statistical power and to simplify the analysis, the month of admission was grouped into 4 seasons:
  - The months December through February classified as (1) winter, March through May as (2) spring, June through August as (3) summer, and September through November as (4) autumn.
- Admission on a week or weekend day:
  - (0) admission on Monday to Friday, (1) admission on Saturday- Sunday
- Disposition of patient from ED was categorized into:
  - (1) routine, (2) transfer to short-term hospital, (5) other transfers, including skilled nursing facility, intermediate care, and another type of facility, (7) against medical advice, (9) admitted as an inpatient to this hospital, (20) died in ED, (98) not admitted, destination unknown, (99) discharged alive, destination unknown (but not admitted)
- Total charges: encompassed the charges in dollars for both settings: ED & hospital.
- Length of stay: reflected how many days the admitted patients stayed in the hospital

Clinical characteristics:

- Chronic conditions body indicator (Yes/No) for different subtypes
- Inpatient procedures (Yes/No)
- Injury diagnosis reported on record and their different types
- Injury severity score was categorized as:
  - (0 -15) Minor trauma, (16 - 75) Major trauma
- More than one injury diagnosis reported on record defined as:
